# Involvement of Inflammation and Its Resolution in Disease and Therapeutics

**DOI:** 10.3390/ijms231810719

**Published:** 2022-09-14

**Authors:** Sebastián Alfaro, Vania Acuña, Ricardo Ceriani, María Fernanda Cavieres, Caroline Ruth Weinstein-Oppenheimer, Carolina Campos-Estrada

**Affiliations:** 1 Escuela de Química y Farmacia, Facultad de Farmacia, Universidad de Valparaíso, Avenida Gran Bretaña, Valparaíso 1093, Chile; 2 Centro de Investigación Farmacopea Chilena (CIFAR), Universidad de Valparaíso, Santa Marta 183, Valparaíso 1093, Chile

**Keywords:** inflammation, resolvins, maresin, tissue engineering, stem cells, chronic wounds

## Abstract

Inflammation plays a critical role in the response to and survival from injuries and/or infections. It occurs in two phases: initiation and resolution; however, when these events do not resolve and persist over time, the inflammatory response becomes chronic, prompting diseases that affect several systems and organs, such as the vasculature and the skin. Here, we reviewed inflammation that occurs in selected infectious and sterile pathologies. Thus, the immune processes induced by bacterial sepsis as well as *T. cruzi* and SARS-CoV-2 infections are shown. In addition, vaccine adjuvants as well as atherosclerosis are revised as examples of sterile-mediated inflammation. An example of the consequences of a lack of inflammation resolution is given through the revision of wound healing and chronic wounds. Then, we revised the resolution of the latter through advanced therapies represented by cell therapy and tissue engineering approaches, showing how they contribute to control chronic inflammation and therefore wound healing. Finally, new pharmacological insights into the management of chronic inflammation addressing the resolution of inflammation based on pro-resolving mediators, such as lipoxin, maresin, and resolvins, examining their biosynthesis, biological properties, and pharmacokinetic and pharmaceuticals limitations, are given. We conclude that resolution pharmacology and advanced therapies are promising tools to restore the inflammation homeostasis.

## 1. Introduction

It is known that the inflammatory process is involved in various physical and mental pathologies such as ischemic heart disease, stroke, diabetes mellitus, cancer, neurodegenerative conditions, depression, and schizophrenia. These diseases are mostly recognized as chronic inflammatory diseases, and they show a high rate of morbidity and mortality worldwide [1]. Inflammation plays a role in wound healing and its undesirable progression to chronic wounds. Therefore, understanding the onset of the inflammatory process in an individual has acquired a potential therapeutic interest. In this review, we focus on describing inflammation as part of the immune response and how pharmacological modulation and advanced therapies demonstrate clinical relevance by early resolution of inflammation. We begin by describing the process of inflammation, using as examples the acute and chronic response to pathogen infection, endothelial cell damage, inflammatory reaction to adjuvants in vaccine formulations, and chronic wounds. We establish that unresolved inflammation is involved in the pathophysiology of chronic diseases, becoming a new therapeutics target. Then, we focused on advanced therapies such as cell therapy and tissue engineering and their contribution to inflammation resolution. Finally, we reviewed the specialized pro-resolving mediators, lipoxins, maresins, and resolvins as we introduced the concept of resolution pharmacology.

## 2. Inflammation

Inflammation is a component of the immune response triggered by injury or infection. It is characterized by both an acute and a chronic phase. Acute inflammation occurs in two phases—initiation and resolution—in which the initiation of inflammation is defined as the activation of the innate immune system against tissue damage (sterile inflammation) or pathogens. On the other hand, the resolution of inflammation is a highly regulated cellular and biochemical process that begins early in the inflammatory response. It is characterized by a 50% decrease in neutrophil infiltration, the synthesis of neutralizing mediators which induce leukocyte apoptosis, clearance of the inflamed site, and tissue regeneration [2,3,4]. A normal inflammatory response is temporary and thus resolves when the inducing agent is eliminated. However, the inflammatory response may become chronic when the inducing event does not resolve and persists over time.

**Figure 1:**
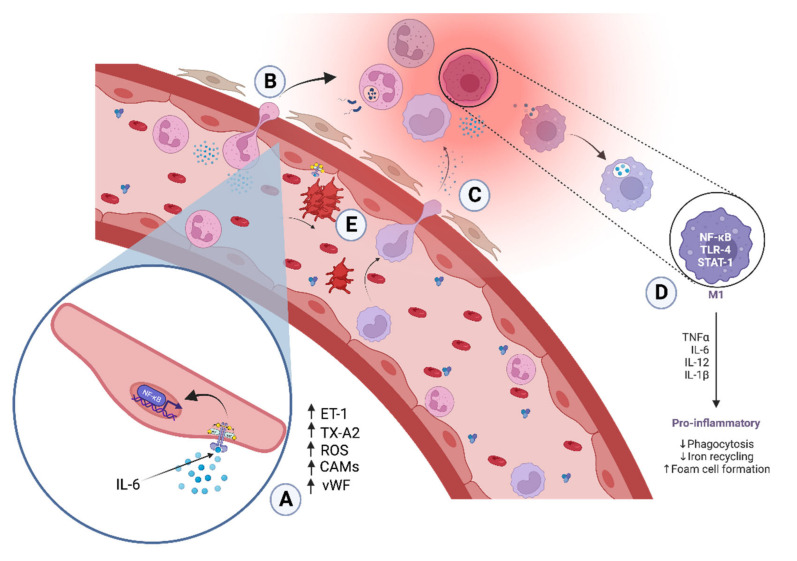
Cellular and molecular events of inflammation. (**A**) Pro-inflammatory cytokines activate nuclear factor kappa B (NF-κB), which in turn increases the expression of endothelin-1 (ET-1), thromboxane A2 (TXA2), reactive oxygen species (ROS), cell adhesion molecules (CAMs), and von Willebrand factor (vWF). (**B**,**C**) Hemodynamic changes and extracellular matrix adhesion molecules (E-CAMs) expression mediate leukocyte recruitment to the extravascular tissues. (**D**) Monocytes differentiate into M1 macrophages type that release pro-inflammatory cytokines. (**E**) Increased expression of TXA2 and vWF favors platelet aggregation (created with BioRender.com, accessed on 30 July 2022).

During acute inflammation, occur vascular and cellular changes. The endothelium changes its phenotype regulating its tone, hemostasis, and permeability. This process is called “endothelial activation” and is characterized by adopting a vasoconstrictive, procoagulant phenotype and increasing its permeability and cell adhesion [5]. In addition, the activated endothelium releases Thromboxane A2 (TXA2) and von Willebrand factor [6], promoting platelet aggregation and coagulation. On the other hand, the cells of the immune system are activated when pathogen-associated molecular patterns (PAMPs) or damage-associated molecular patterns (DAMPs) are recognized by pattern-recognition receptors (PRRs), triggering the release of pro-inflammatory signals [7], which increase the expression of cell adhesion molecules (CAMs), leading to leukocyte attraction and adhesion through mechanisms involving the activation of nuclear factor kappa B (NF-κB), as shown in Figure 1.

Acute inflammation is a natural protective mechanism of the host in response to injury or invading pathogens. Normally, the immune response eradicates the infection; consequently, the inflammatory phenomenon tends to be resolved through endogenous molecules known as specialized pro-resolving (SPM) produced mainly by macrophages and neutrophils [4].

The resolution of inflammation is a highly regulated cellular and biochemical process that begins during the first hours of the inflammatory response. The resolution of inflammation is characterized by (i) a 50% cessation of polymorphonuclear neutrophils (PMN) recruitment, which involves metabolic changes in the synthesis of neutralizing mediators that induce leukocyte apoptosis, clearance of the inflamed site and tissue regeneration through phagocytosis; (ii) a decline in the number of vessel wall macrophages and conversion to the M1 phenotype; (iii) a reduction in pro-inflammatory cytokines and growth factors expression; (iv) a decline in the vascular smooth muscle cells (VSMC) proliferative index; and (v) regeneration of an intact endothelium and the initiation of matrix remodeling [2,4].

The inflammatory response is often temporary. However, when these events do not resolve and persist over time, the inflammatory response becomes chronic, which may become pathological with devastating consequences. During the past decade, an abundance of new evidence highlighted the importance of inflammation in the development of chronic pathologies such as neurodegeneration, asthma, cancer, diabetes, cardiovascular diseases such as atherosclerosis or Chagas disease, inflammatory bowel disease, and rheumatic disease [8,9,10].

## 3. Inflammation and Associated Diseases

### 3.1. Endothelial Inflammation in Atherosclerosis

Atherosclerosis results from an unresolved inflammatory process that involves mediators and key inflammatory events such as the oxidation of lipoproteins, endothelial activation, leukocyte recruitment, and the proliferation of VSMCs. In fact, endothelial activation provokes flow changes, mechanical stress, and imbalance between nitric oxide (NO·), prostacyclin (PGI2), and endothelin-1 (ET-1), as well as the expression of cell adhesion molecules (CAM-1, intercellular adhesion molecule 1 (ICAM-1), E-selectin), which, when combined with the release of pro-inflammatory cytokines (interleukin-1 β (IL-1β), IL-6, tumor necrosis factor α (TNFα), and monocyte chemoattractant protein-1 (MCP-1)), triggers the recruitment of neutrophils and monocytes to the damaged site [11,12]. Monocytes will differentiate into macrophages, releasing ROS, growth factors, proteases, and internalize oxidized low-density lipoprotein (LDL) particles as well as other deposited debris, forming foam cells that will perpetuate endothelial dysfunction [13,14]. Proinflammatory activation of monocytes has been correlated to mitochondrial DNA mutations in patients with asymptomatic atherosclerosis whose monocytes were isolated. This would suppose an additional hypothesis of atherogenicity and its associated inflammatory processes [15]. IL-1β can stimulate the production of growth factors (fibroblast growth factor (FGF), transforming growth factor β (TGF-β), platelet-derived growth factor (PDGF)) that rapidly activate the proliferation of fibroblasts and change the phenotype of VSMCs from a basal contractile state to a differentiated phenotype. Differentiated VSMCs proliferate, migrate, and synthesize extracellular matrices (collagen and elastin), expressing pro-inflammatory signals. These events establish the maturation and progression of the atherosclerotic plaque to a fibrotic lesion that hardens the arterial wall [3]. Finally, chronic inflammation causes vascular damage, which gradually leads to a reduction in or narrowing of the lumen of the blood vessels, generating ischemia at the vascular level [16,17].

Interestingly, not all individuals resolve acute inflammation, and even those individuals who manifest high levels of inflammatory markers do not always develop pathology. Understanding the endogenous and environmental mechanisms that led to chronic damage is crucial to be able to propose diagnostic and therapeutic strategies.

### 3.2. Pathogens-Anduced Inflammation

#### 3.2.1. Bacterial Sepsis

Sepsis is defined as an organ dysfunction caused by an uncontrolled infection that synergizes with an exacerbated inflammation. Bacterial sepsis can be caused by *Staphylococcus aureus*, *Escherichia coli*, or *Pseudomonas aeruginosa*, reaching a death rate of 20% [18,19]. Several studies have associated sepsis with exacerbated inflammation even with cytokine release syndrome (CRS); healthy individuals have regulatory mechanisms to control inflammatory responses, but advanced age, comorbidities, and suppressed adaptive immunity enable organ injury and death [20,21,22]. The molecular alterations in sepsis onset when bacteria PAMPs as lipopolysaccharide (LPS) are recognized by PRR such Toll-like receptor 2 (TLR2) or Toll-like receptor 4 (TLR4) that activate myeloid differentiation factor 88/IL-1 receptor-associated kinase 1/tumor necrosis factor receptor-associated factor 6 (MyD88/RAK/TRAF6) signaling pathways to induce activation of NFκB, and subsequently, excessive release of pro-inflammatory cytokines such interleukin-1 (IL-1), interleukin-6 (IL-6), interleukin-17 (IL-17), and TNFα generated hyperinflammation. Interestingly, studies have revealed that TNFα level increases in non-survivors compared to survivor patients, which could be a util biomarker for stratifying a hyperinflammatory patient [23,24,25]. In addition, complement activation, redox imbalance, mitochondrial dysfunction, and immune suppression culminate in multi-organ failure [18]. However, conventional therapies focus on inhibiting acute inflammation but not on the resolution of inflammation. Interesting resolution targets will be discussed later.

#### 3.2.2. Viruses and Immune Response

As we witnessed in the last two years, virus infection can have devastating consequences for mankind. Virus is recognized by innate immune receptors, known as toll-like receptors (TLR), some of them being transmembrane while others are cytosolic, allowing the sensing of both extra and intracellular viruses [26]. The virus–TLR interaction also displays a role in the adaptative immune response by activating the expression of co-stimulatory molecules and also upregulating the major histocompatibility molecules (MHC) on dendritic cells [27,28]. Thus, the purpose of TLR–virus interaction is to control the infection; however, it has been shown for virus-like SARS-CoV-2 that the TLR pathways are involved in the damage to the host by triggering inflammation and tissue destruction [28]. Thus, SARS-CoV-2 interacts with Toll-like receptors (TLRs), activating the inflammasome, which results in IL-1 production. The latter triggers IL-6 secretion, which has been implicated in the physiopathology of SARS-CoV-2 [29]. Additionally, a reduction in the expression of Interferon (IFN) receptors has been reported as a cause of the inflammatory systemic response observed in COVID-19 [30].

The severity of COVID-19 has been associated with higher plasma levels of pro-inflammatory cytokines, such as IL-1, IL-6, IL-8, and TNFα, among others [29]. A meta-analysis using logistic regression concluded that IL-6 and interleukin-10 (IL-10) together displayed prognostic potential for the severity of the disease, exhibiting a nearly 100% specificity and 83.3 sensitivity for classifying severe and non-severe patients [31]. Elevated serum IL-6 has also been reported as a marker of disease severity [32]. It is especially interesting to analyze the interplay between IL-6, an important cytokine for mucosal immunity, and viral lung infection. Hence, IL-6 is an important player in the initiation of the adaptative immune response by stimulating CD8 lymphocytes, B cells, and Treg; and favoring the survival of phagocytic neutrophils. However, IL-6 can also counterbalance Th differentiation towards the Th2 and Th17 phenotypes, which mediates tissue injury facilitating basal membrane and matrix disruption, increasing vascular permeability, and causing edema. The Th2 phenotype through IFN-γ secretion will decrease the NK and CD8 cytolytic function. Th17 cells secrete IL-17A, known to up-regulate Bcl-2, which results in the inhibition of apoptosis of infected cells [33]. IL-10, the other COVID-19 named before as a COVID-19 prognostic cytokine, has been associated with an impaired function of mucosal-associated innate T cells (MAIT), which are innate-like T cells with a semi-invariant T cell receptor (TCR). The MAIT cells participate in the antibacterial [34] and antifungal immune response [35], have been implicated in HIV-1 chronic infection [36], and more recently, have been related to COVID-19 severity [37]. Moreover, MAITs dysfunction was associated with the severity of the disease and the presence of bacterial or fungal co-infection [38]. In addition, it was shown that monocytes from severely diseased COVID-19 patients were suppressive for MAITs [36].

In vitro experiments showed that the suppressive effect of monocytes on MAITs were mediated by IFN-α-induced IL-10. These authors also showed that IL-10 mediated a reduction in human leukocyte antigen—DR isotype (HLA DR) expression on monocytes, which is an immune-suppressive effect [38]. Even though SARS-CoV-2 is a recently known viral infection and more studies are required to fully understand its physiopathology, it is clear so far that COVID-19 is another example of a disease caused by aberrant immune overreaction.

#### 3.2.3. Parasitic Infection in Chagas Disease

Chagas disease is characterized by chronic inflammation caused by a parasite infection by *Trypanosoma cruzi* (*T. cruzi*). The parasite-infected nucleated cells induce endothelial activation, prior to the initiation of the inflammatory process, through the release of ET-1 that leads to vasoconstriction and increased cell permeability [39]. In addition, the activated endothelium releases TXA2 and von Willebrand factor, promoting platelet aggregation and coagulation. On the other hand, infection of endothelial cells and macrophages by *T. cruzi* causes increased expression of pro-inflammatory cytokines, ROS, and CAMs, leading to leukocyte attraction and adhesion through mechanisms involving the activation of NF-κB [40,41]. In fact, previously, we confirmed through transcriptomic analysis that several pathways involved in the biological response to parasitic infection were upregulated in the early stages of intracellular infection. As expected, immune-response-related genes increased their expressions such as type II interferon signaling, interferon alpha/beta (IFN-α/β) signaling, Toll-like receptor signaling [42], extracellular matrix adhesion molecule (E-CAM) expression, and interleukin 6 signal transducer. We found that benznidazole and simvastatin have an anti-inflammatory effect mediated by the production of 15-epi-lipoxin A4 (15-epi-LXA4) and block of the activation of NF-κB, as we reported previously [41]. Interestingly, we found that the benznidazole treatment induced the upregulation of the Notch pathway; this signaling is involved in the regulation of vascular inflammation, and cardiac remodeling [43].

*T. cruzi* infection, whereby its persistence in the heart tissue causes persistent inflammation without resolution, triggers irreversible structural changes associated with cardiovascular remodeling that results in loss of cardiac function [44].

Both pathogen-induced and sterile inflammation require a balance between pro- and anti-inflammatory mechanisms. The action of the immune system, which includes the complement system, the innate immune system (IBS), and the specific immune system (SIE), is crucial to define whether the inflammatory process is capable of being resolved in an acute stage or if it will persist as an inflammatory chronic status. On the other hand, the etiology of inflammation may also be induced by molecules that activate immune responses such as adjuvants in vaccines. In fact, this mechanism is useful to trigger an immune response against antigens. Adjuvants are chemically diverse molecules that stimulate a strong immune response against an antigen, improving the potency, durability, and quality of the immune response elicited by them.

### 3.3. Adjuvants-Induced Inflammation

Adjuvants in vaccine formulation allow us (i) to decrease the amount of antigen, (ii) reduce the number of doses, (iii) induce a rapid protective response, and (iv) increase the rate of seroconversion in special populations. Ideally, an adjuvant should maximize the immunogenicity without compromising the tolerability or safety of the vaccine. However, few adjuvants have been approved for human use as it has been difficult to find the balance between immunogenicity and immunotoxicity [45,46,47].

Adjuvants that act as immune potentiators mimic the signals induced by pathogens, activating the innate and adaptive immunity by the interaction between PAMPs and components released from damaged tissues called DAMPs. These molecules are detected by receptors on immune cells, known as PRRs, such as TLRs and cytosolic inflammasome-stimulating Nod-like receptors (NLRs). The NLR inflammasome is a multiprotein complex which, upon activation, has the potential to regulate the differentiation of T-helper subsets [48,49]. The recognition of these signals by PRR on macrophages and dendritic cells (DCs) leads to the activation of NF-κB and interferon regulatory factor 7 (IRF7), via myeloid differentiation factor 88/domain-containing adaptor protein (MyD88/TIRAP) or TIR-domain-containing adapter-inducing interferon-β/TRIF-related adaptor molecule (TRIF/TRAM), to express inflammatory cytokines TNFα, IL-1, IL-6, among others, chemokines, endothelial adhesion molecules (E-selectin, ICAM-1, vascular cell adhesion molecule-1 (VCAM-1) and platelet endothelial cell adhesion molecule-1 (PECAM-1), co-stimulatory molecules (cluster of differentiation 80 (CD80), cluster of differentiation 83 (CD83) and cluster of differentiation 86 (CD86)), and IFN. Expression of these molecules creates a local pro-inflammatory environment, where phagocytes are recruited, leading to the activation of the complement cascade and chemo-attraction of the effector cell-mediated adaptive immune response [50,51,52,53].

Currently, vaccine development has evolved rapidly due to the pandemic, and promising candidates have emerged as adjuvants to COVID-19 vaccines. The new-generation adjuvants attempt to add an immune-potentiator, and some of these adjuvants have already been licensed for human vaccines such as Monophosphoryl Lipid A (MPL) and QS-21. MPL is a TLR4 agonist purified from endotoxins, induces the maturation of antigen-presenting cells (APCs), and triggers the release of cytokine-enhanced humoral and cellular immune responses. QS-21 is a fraction purified from a saponin extract derived from the bark of *Quillaja Saponaria*, which induces strong cellular humoral and T-cell responses. QS-21 is one of the most potent adjuvants known; in fact, it has been studied as an alternative to alum when strong cellular responses are required for a particular vaccine. This is because it has exhibited an excellent adjuvant property for a range of antigens and has proven to be more potent in activating a broader adaptive immune response [54]. However, few adjuvants are licensed for human use because of their adverse effects and toxicity. Therefore, there is an antagonism between the capacity of the immune system to respond to various stimuli and the exaggerated reaction of the host that can cause severe damage and even disease if it remains uncontrolled. In previous results, we have shown that adjuvants such as MPL and QS-21 can activate an early inflammatory response, triggering a cytokine cascade. In addition, both demonstrate immunostimulatory activity in combination. Finally, QS-21 and MPL alone can activate NFκB; both adjuvants in combination overexpress neurogenic locus notch homolog protein 1 (NOTCH-1) [55]. This would explain, at least in part, its immunogenic potency, which supports use in vaccines such as the Novavax COVID-19 vaccine with matrix M-adjuvant containing saponin, recently approved by the Food and Drug Administration (FDA) [56].

#### Uncontrolled Inflammation Caused by Adjuvants in Vaccines

The immunological action of adjuvants can be associated with local and/or systemic reactions. Local adverse events are frequent side effects of vaccines and include pain, inflammation, abscess formation, ulceration, lymphadenopathy, lump at the injection site, redness, pruritus, and hematoma. After inoculation, the first effect is a transitory inflammatory reaction, driven by chemokines such as Chemokine C-X-C motif ligand-1 (CXCL1), Chemokine C-X-C motif ligand-2 (CXCL2), Chemokine C-C motif ligand 3 (CCL3), Chemokine C-C motif ligand 4 (CCL4), Chemokine C-X-C motif ligand-5 (CXCL5), Chemokine C-C motif ligand 8 (CCL8), and Chemokine C-C motif ligand 20 (CCL20), which promote neutrophil recruitment and migration. Subsequently, macrophages release anti-inflammatory cytokines, and if the inflammation persists, “off-target” effects are generated [57,58] including cytolytic effects, Arthus reaction, recall urticaria, cutaneous lymphoid hyperplasia, and lymphadenitis. This reaction has been observed with saponins (QuilA or QS-21), which interact with cell membranes, inducing cell lysis and direct local production of Chemokine C-C motif ligand 2 (CCL2) and CXCL1. Granuloma formation may appear when excessive amounts of oil emulsions are injected at a single site. It begins with the migration of macrophages due to a persistent inflammatory stimulus followed by the accumulation of CD4+ T cells and macrophages. Then, the infiltrating cell population decreases, allowing local fibrosis to form [59,60].

Systemic effects are fever, congenital anomaly/birth defect, anaphylaxis, life-threatening conditions, and death. Toxic systemic effects after adjuvant administration are a consequence of the hyper-activation of immunological mechanisms induced by the adjuvant formulation. These effects are often mediated by the release of IL-1β, TNF-α, interferon β (IFN-β), interferon γ (IFN-γ), and IL-6 cytokines. Some important systemic effects include acute phase response, vascular leak syndrome, induction or worsening of autoimmunity, allergy, embryo-fetal immunotoxicity, and modification of hepatic metabolism associated with the release of interleukins. On the other hand, IL-1, interleukin-2 (IL-2), IL-6, TNFα, TGF-β, and IFN are involved in modulating a decrease in the expression of cytochrome P450 1A (CYP1A), cytochrome P450 2B1/2 (CYP2B1/2), and cytochrome P450 3A4 (CYP3A4) isoforms subfamily. Given this modulation of enzymatic expression, it is important to consider differences in expression of cytochrome (CYP) polymorphisms among individuals receiving the vaccination as this may confer special susceptibility to both the immunogenicity and toxicity of adjuvants [48,61,62]. Adverse events are usually associated with a type of adjuvant, although there is limited information on the toxicity induced by the adjuvants themselves as toxicity studies are mainly performed with whole formulations. Thus, it is difficult to predict the exact profile of their side effects because both antigens and adjuvants can contribute to the toxicity.

### 3.4. Wound Healing

#### 3.4.1. Phases of Wound Healing

As the outer barrier, the skin is the organ most challenged by external stress factors that can damage its integrity [63]. However, the skin has a healing capacity and, upon damage, can trigger mechanisms to replace the missing cellular structures and tissue layers. The skin wound healing process can be divided into four main phases: hemostasis, inflammation, migration and proliferation, and remodeling [64,65]. This process involves various inflammatory cells, chemokines, cytokines, matrix molecules, and nutrients delivered to the wound site from adjacent tissue [66].

Hemostasis is the first phase of skin wound healing, which occurs immediately after injury. The lesioned blood vessels contract and the leaked blood coagulates to stop local hemorrhage. This is achieved by platelet activation and aggregation, which results in the formation of a fibrin clot, generating a temporary scaffold structure for the migration of leukocytes, keratinocytes, fibroblasts, and endothelial cells and as a reservoir of growth factors [67]. During the inflammatory phase, vascular permeability increases with vasodilation, allowing neutrophils and monocytes to localize to the wound site [68]. These are attracted by many inflammatory cytokines produced by activated platelets, endothelial cells, and degradation products of pathogenic agents [66]. The role of the neutrophils is crucial to clearing the initial break-in of contaminating bacteria [69]. Furthermore, they release pro-inflammatory cytokines, activating other cells such as fibroblasts and keratinocytes [70]. Approximately 48 h after the injury, the monocytes are differentiated into macrophages. The macrophages phagocytose, digest tissue debris, and secrete growth factors and cytokines such as TGF-β, basic fibroblast growth factor (bFGF), PDGF, and vascular endothelial growth factor (VEGF), which promote cell proliferation and the synthesis of extracellular matrix (ECM) molecules by resident skin cells [71]. Approximately 3–10 days after wounding, the proliferative and migratory phase starts. It is characterized by fibroblast migration and the deposition of ECM proteins such as hyaluronan, fibronectin, proteoglycans, and procollagens I and III, generating granulation tissue [72]. Then, fibroblasts change to their myofibroblast phenotype, allowing wound contraction and decreasing the lesioned tissue area [73]. Furthermore, the epithelialization process occurs by keratinocytes originating from the wound edges and epithelial stem cells from hair follicles or sweat glands, generating the newly stratified epidermis [74]. This phase also includes angiogenesis, which involves the sprouting of wound edge capillaries followed by their invasion into the site of damage. After a few days, a microvascular network is apparent throughout the wound, which provides nutrients and oxygen to the growing tissues and aids in the formation of granulation tissue [75]. The remodeling phase begins two-to-three weeks after the onset of the lesion and can last for one year or more. During this phase, the formation of granulation tissue stops, and collagen III, which was produced in the proliferative phase, is replaced by collagen I, developing final scar tissue formation [76]. If the above-described stages of wound healing do not occur in a timely manner, then chronic wounds will develop.

#### 3.4.2. Chronic Wounds

A chronic wound is defined as one that does not undergo the four phases of wound healing in a timely and orderly manner, failing to heal [77]. Unfortunately, this condition is very common. It is estimated that 1 to 2% of the population will experience a chronic wound during their lifetime in developed countries [78]. Several causes, such as infection, hypoxia, necrosis, or exudate and pro-inflammatory cytokines, can prolong the phases of wound healing [79,80]. The underlying causes might be naturopathy, pressure, arterial or venous insufficiency, burns, and vasculitis and are more prevalent in elderly, obese, and diabetic patients [72,81,82]. It is known that both aging and diabetes make skin more prone to injuries and that once a wound occurs, several changes aggravate the possibilities of normal healing [77]. One of them is cellular senescence, with a reduced proliferative capacity, a hypersecretory phenotype of pro-inflammatory cytokines and tissue degrading enzymes that has been reported on fibroblasts from diabetic foot ulcers, venous ulcers, and pressure ulcers [82,83,84]. Dermal fibroblast senescence has been related to autophagia suppression mediated by the miR-1299/ARG/ARL1 axis, which, interestingly, is antagonized by the pro-resolving resolvin D1 (RvD1). The pro-resolving RvD1 downregulates arginase (ARG) expression, induced by H2O2 in dermic fibroblasts, causing a recovery in the number of cells undergoing autophagia [81,85].

Thus, several factors are involved in disproportionate inflammation in chronic wounds, associated with an excess of immune cell infiltration and pathological functions, which collectively prevent the shift from inflammation to resolution and also make the wound prone to infection. This can bring the wound into a vicious circle of infection and inflammation [81].

Generally speaking, it is recognized that the availability of better treatments would contribute to the healing of a proportion of chronic wounds; nevertheless, there are some that will remain unhealed with the available standard care, highlighting the need for a better understanding of the molecular and cellular basis of wound healing [81,82].

Overall, the understanding of the processes involved in wound healing will allow the restoration of the balance of the immune response within the wound. A particular attention has been suggested for the consideration of the role of non-immune cells in an extracellular matrix [86]. The latter might be considered as the fundamentals of advanced therapies for wound healing. Such knowledge has brought about novel therapeutic approaches known as advanced therapies, which will be described next.

## 4. Therapeutics

### 4.1. Conventional Therapies

As stated above, it is well established that inflammation has a key role in vascular damage, and inflammatory mediators have been widely studied both as causal agents as well as biomarkers associated with cardiovascular risk. Thus, resolving inflammation is a potential therapeutic target for reducing cardiovascular risk.

Several clinical studies have shown that drugs commonly used in cardiovascular diseases (CVDs), such as statins, help in the primary and secondary prevention of cardiovascular events. This beneficial effect is not only explained by their ability to lower cholesterol levels but also because of their anti-inflammatory effects [87]. A recent meta-analysis has confirmed that most of the beneficial effects of statins are independent of their lipid-lowering action. Several studies show that statins decrease C-reactive protein (CRP) levels [88,89,90]. The clinical trials Cardiovascular Inflammation Reduction Trial (CIRT) and Canakinumab Anti-inflammatory Thrombosis Outcomes Study (CANTOS) have researched this hypothesis with promising results. The use of methotrexate at low doses combined with standard care caused a reduction in IL-6 and CRP levels in patients with chronic atherosclerosis in CIRT [91]. Moreover, a reduction in IL-6, CRP and a significantly lower incidence of recurrent cardiovascular events in patients with coronary artery disease (CAD) with persistent inflammatory states were reported in CANTOS [92]. This clinical evidence strongly supports the hypothesis that providing anti-inflammatory treatment for patients with chronic CVD has vasculo-protective benefits. In addition, these studies demonstrated the causal role of inflammation in atherosclerosis and how these drugs offer additional advantages over other anti-inflammatory agents [91,93,94,95].

Traditional anti-inflammatory agents have limited efficacy on inflammation in atherosclerosis. Some anti-inflammatory drugs can cause toxicity in the endothelium with the risk of thrombosis, and others can cause suppression of the immune system, generating a risk of infections [2,96]. Moreover, none of these drugs avoid the vascular damage induced by surgical interventions, and most importantly, these drugs are not able to resolve and repair endothelial damage. Furthermore, the elucidation of pathways and mediators involved in the resolution phase of the inflammatory process has generated a change in the paradigm of a passive resolution process.

### 4.2. Advanced Therapies for Wound Healing

An acute wound in stable patients should not impose a threat to its proper healing if adequate care is given. However, chronic wounds are a challenge, in which the underlying medical conditions are not easy to find [97]. Debriding is a good opportunity to transform a chronic wound into an acute one by removing necrotic, infected, or hyperkeratotic tissue. The latter is followed using saline or antibacterial solutions and a proper wound dressing [98]. However, the health quality of life of patients with chronic wounds is poor and the cost of treatment is elevated [99]. Thus, it is not always that simple, and novel therapies are urgently needed that address more than the secondary consequences of chronicity [72,81].

A chronic wound is treated with cleaning solutions, debridement and covered with wound dressings and bandages. The dressings might be films, gauze, hydrogels, hydrocolloids, foams, silver, or alginates, some of which will have the capacity to absorb exudates. Compression is used when poor circulation is causing the wound. For extensive wounds, autografts are utilized [97]. It has been shown that some washing solutions containing surfactants will reduce microbial biofilms and will therefore contribute to the control of inflammation [100]. Debridement and absorption of exudates also contribute to anti-inflammation by removing the triggering stimulus. Nevertheless, these therapeutic approaches have not been completely successful. Thus, advanced therapies are needed to resolve inflammation in chronic wounds.

#### 4.2.1. Cell Therapy

Mesenchymal stem cells (MSCs) have been the most intensively investigated to develop cell therapies for wound healing. These are multipotent stem cells that in theory differentiate into cells belonging to their same embryonic origin [81]. The International Society for Cell Therapy has established criteria to define these cells: (a) they are adherent to plastic under conventional cell culture conditions; (b) more than 95% of the population is positive for the markers Cluster of Differentiation 105 (CD105), Cluster of Differentiation 90 (CD90), and Cluster of Differentiation 73 (CD73), and 98% of the population lacks hemopoietic markers, such as Cluster of Differentiation 34 (CD34), Cluster of Differentiation 14 (CD14), Cluster of Differentiation 11b (CD11b), Cluster of Differentiation 79 α (CD79α) and Cluster of Differentiation 19 (CD19) and HLA DR; and (c) they exhibit the capacity to differentiate into osteoblasts, chondroblasts, and adipocytes under defined cell culture conditions [101].

The stem-cell mechanisms for wound healing include the provision of the cells per se to colonize the wound bed, the production of extracellular matrix components, and wound healing factors. Moreover, these cells also contribute indirectly to the healing process by modulating the immune system and thus activating fibroblasts to direct resident stem cells to the wound site for tissue reconstruction [102].

In addition, MSCs can inhibit the inflammatory phase, accelerating the healing process in chronic wounds by controlling the macrophage response. MSCs promote polarization from M1 towards a M2 phenotype, which exhibits anti-inflammatory activity and suppresses the production of TNF-α during wound healing [103]. Moreover, the tumor necrosis factor-α (TNF-α)-stimulated gene 6 (TSG-6) generated by MSCs decreased the release of TNF-α from macrophages and induced a low TGF-β1/transforming growth factor β3 (TGF-β3) ratio, decreasing the production of fibrotic tissue [104]. It was recently reported that dental pulp-derived stem cells exhibited an anti-inflammatory behavior when stimulated by bioactive agents, driving macrophages towards the M2 phenotype and promoting faster in vitro wound healing [105].

Interestingly, some authors describe MSCs as a “sensor and switcher of the immune system”; thus, depending on the state of the immune system, it could turn it on or off. This might explain the powerful tool that stem cells might imply to perform cell therapy for wound healing. These cells could, on the one hand, sense danger or pathogens through TLR, activating the immune system, and on the other hand, restrain inflammation to move the process of wound healing to a process that would resemble the acute wound healing process. An important component of the sensing properties of MSCs occurs through TLRs, TRL4 activation being a trigger for pro-inflammatory effects and TRL3 being an inducer of anti-inflammatory actions. In addition, there are several MSCs regulators, such as IFNγ, that induce the expression of the immune inhibitors PDL-1 and PDL-2 on these cells. The MSCs on their immune-suppressive state downregulate MHC I and MHC II and co-stimulatory molecules. An important number of mediators have been shown to be upregulated by the anti-inflammatory MSC, such as IDO, PGE2, iNOS, TGF-β, CCL-2, IL1RA, among others, as well as some immune cells, including dendritic cells, momocytes/macrophages, B cells and T cells. For T cells, MSCs have been associated to a Th2 polarization and inhibition of Th17 differentiation [106].

Recently, the therapeutic potential of MSCs-derived exosomes has been reported [107]. Exosomes are nano-sized extracellular vesicles (30–150 nm) secreted from the endosomal membrane of eukaryotic cells, allowing the communication between cells and intracellular signaling [43,108,109,110]. They contain messages, including proteins such as cytokines and growth factors, lipids, miRNA (microRNA), and DNA fragments [111,112]. In addition, studies have proved that the MSC exosomes modulate inflammation in skin wounds, promoting M2 polarization of macrophages, decreasing TNF α, TLR4, NF-κB, NOX1, NOX4, IL-6, IL-1β, and p-p65 and increasing the IL-10 level, thus accelerating the wound healing. Additionally, the MSC exosomes stimulate the production of essential factors in the process of granular tissue generation, such as N-cadherin, cyclin 1, PCNA, collagen I/III, elastin, fibronectin, and angiogenesis, by increasing VEGF expression [110,113,114,115,116].

Several tissues provide stem cells for cell therapy, such as bone marrow, adipose tissue, skin, and dental pulp, umbilical cord, Wharton’s jelly, placenta, and others [99]. These cells secrete cytokines and trophic factors, and for [117] mesenchymal cells isolated from the human dental apical papilla, it was shown that the secretion of transforming growth factor β3 (TGFβ-3), a pro-healing cytokine, was higher on cells grown in three-dimensional materials. Interestingly, the results suggested that more relevant than the material itself was the 3D growth of the cells, which triggered the growth factor’s secretion [101,117]. These observations have set the fundamentals of a relatively new basic clinical area of research called tissue engineering, which emerged about thirty-five years ago [118] The aim of tissue engineering is to produce biological materials for tissue regeneration [119].

#### 4.2.2. Tissue Engineering

The transplant of free stem cells on the diseased skin has several drawbacks; it is believed that 1% of the cells will survive 24 h, which is attributed to the lack of extracellular matrix and the hostile environment of the wound bed. Moreover, a recent three-year follow-up study of a clinical intervention in which diabetic ulcers were treated with topical application of MSCs showed that even though the therapy was safe in the long term, the efficacy was limited since the skin parameters such as the collagen density and epidermal thickness were altered compared with the normal skin structure [120]. For this, it has been suggested that it is better to provide the stem cells within a scaffold made of a biomaterial that mimics the extracellular matrix and provides mechanical resistance [121].

Currently, there is no skin substitute that completely mimics the real tissue, the desired attributes being easiness to handle and to apply on the wound, serving as a barrier function, adhesivity, proper physical and mechanical properties, degradability, sterilizable, non-toxicity, and non-antigenicity, eliciting minimal inflammatory activity, ideally being pro-angiogenic, and cost-effectiveness [119].

Traditional wound dressings are passive in contrast with biomaterial-based wound dressings which are aimed to provide some of the above-mentioned properties [122]. Several tissue engineering devices have been developed exhibiting in vitro and in vivo cell biocompatibility [123,124,125]. A cellular amniotic membrane was shown to be a natural scaffold to vehicularize umbilical cord MSCs, showing biocompatibility and efficacy on wound closure after being tested on a clinical trial on diabetic ulcers, in which three courses of treatment drove a mean percentage of recovery of 96.7% with a self-report of a decline in pain [126].

**Figure 2:**
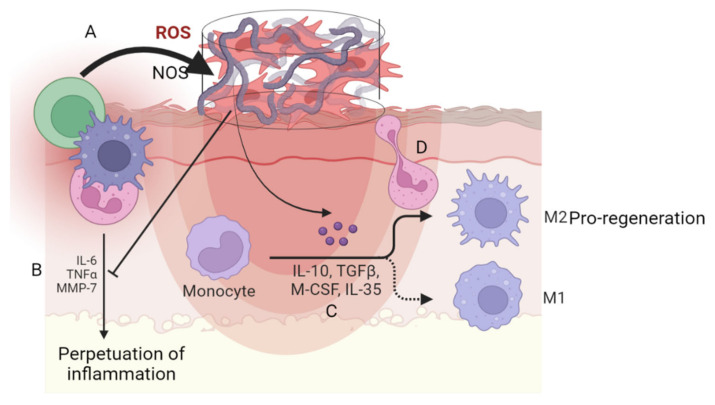
Regulation of the inflammatory response by tissue engineering devices. (**A**) Represents the capacity of biomaterials to scavenge oxidative species. (**B**) The inhibition by MSCs of the pro-inflammatory cytokines. (**C**) The polarization of macrophages towards the M2 phenotype. (**D**) The blockade of immune cell migration (created with BioRender.com, accessed on 30 August 2022).

Interestingly, biomaterials can not only provide a vehicle for the cellular component of advanced chronic wound therapies but also might contribute to the anti-inflammatory properties of the device, as summarized in Figure 2. It has been reported that some biomaterials capture pro-inflammatory mediators and block cellular interactions associated with the inflammatory process [127]. Among the mediators that are scavenged by biomaterials, the capture of damaging reactive oxygen species improves the function of MSCs for skin restoration [128].

The most used biomaterials are poly(glycolic acid), poly(lactic acid), poly(lactic-co-glycolic acid), collagen, chitosan, gelatin, polycaprolactone, hyaluronic acid, and silicone [129]. For certain biomaterials, an effect on inflammatory processes has been reported. Thus, a chitosan hydrogel containing a Chinese medicinal herb showed the inhibition of the miR-29 inflammatory pathway accompanied by a reduction in M1 macrophage polarization on a diabetic rat wound model [130]. Chitosan hydrogels loaded with MSCs have also shown the capacity to reduce inflammatory cell infiltration during in vivo wound healing [131]. For hyaluronic acid, Alemzadeh conducted an in vivo assay using a burn model of injury in rats and a system composed of acellular dermal matrix plus hyaluronic acid hydrogel seeded with adipose-derived MSCs. Not only did wound closure occur faster for the tissue-engineered device but markers of inflammation, such as IL-1β and TGF-β, also showed reduced expression at 7 days post-administration. These results were consistent with the histopathological analysis of the wounds, showing a lower presence of inflammatory cells for the hydrogel containing MSCs treated wounds [54]. Another research involving an in vitro skin raft thermal injury model used a 3D core/shell scaffold composed of MSCs and endothelial cells, showing that pro-inflammatory cytokines IL-6 and IL-1β were statistically reduced at day 0 and 1, respectively, compared to the burned cultures with no treatment [132]. Among synthetic polymers used for building cell-seeded scaffolds [133], a PEG-polyurethane scaffold loaded with bone-marrow-derived MSCs also showed, in a murine wound mouse model, a decreased expression of pro-inflammatory cytokines, such as IL-1β, IL-8, and TNF-α, as well as an increase in the levels of anti-inflammatory cytokines, such as IL-10 and interleukin-13 (IL-13) [134].

Some experts have suggested that MSCs should be “licensed” with a pro-inflammatory stimulus in order to fully exert their immunomodulatory role [133]. Thus, an alginate collagen hydrogel loaded with MSCs and heparin-coated beads loaded with IFN-γ, as licensing stimulus was in vitro analyzed, showing the immunosuppressive behavior of licensed MSCs through upregulation of IDO-1, Gal-9, and PTGS2, whereas IL-10 and IL-1RN were unresponsible to MSCs licensing [135].

Without any doubt, 3D-printing revolutionized the tissue engineering field. Thus, the extrusion technique provided, among others, a polydopamine-modified bioceramic with a mussel-inspired nanostructure showing in vitro production of immunoregulatory mediators of pro-inflammatory function, such as cyclooxygenase-2 (COX-2), PEG-2, and TSG-6; however, the conditioned media of the cultured MSCs within the scaffold exerted an M2 polarization effect on cultured macrophages [136].

In the pharmaceutical market, certain tissue-engineering products have been approved for wound treatments. Thus, Apligraf is an FDA-approved cell therapy for wound healing consisting of allogeneic fibroblasts and keratinocytes in bovine collagen. Its first approved indication is for chronic cutaneous ulcers and then the same product was approved with the trade name of Gintuit for the treatment of a surgically created vascular wound bed in the treatment of mucogingival conditions in adults. Statagraph is another cell product, which is approved for deep partial skin thermal burns; it is made of allogenic keratinocytes and dermal fibroblasts on murine collagen as a scaffold [137]. Even though there is only one FDA-approved tissue engineering device, specifically approved for the treatment of chronic wounds, clinical research in this area shows a variety of strategies. A search on the clinicaltrials.gov platform, which includes most of the current clinical trials around the world was performed crossing the terms stem cells and chronic wounds, allowing us to build Table 1. Representative clinical trials are presented in this table, showing that this is an area of intense research to find the best strategy to use advanced therapies for chronic wound healing, achievable by investigating the type of cells, dosage, and route of administration [108].

### 4.3. Resolution of Inflammation as a Novel Therapeutic Target

Resolution is indeed an active process that is highly regulated and orchestrated by endogenous mediators, such as lipoxins, resolvins, protectins, and maresins. Collectively, these mediators are known as SPMs [107,138,139]. The discovery of these new chemical entities with the ability to resolve inflammation has opened a new research field known as resolution pharmacology. There are different pharmacological strategies to manage the inflammatory processes in these inflammation-mediated diseases; for example, the use of corticosteroids in asthma has been widely described [9], as well as immunotherapies with methotrexate, leflunomide, or hydroxychloroquine and monoclonal antibodies for rheumatic disease [10]. At the same time, the inflammatory pathophysiology associated with Chagas disease has allowed progress in the knowledge of drugs regularly used in cardiovascular pathologies and their role in the resolution of inflammation. This is the case in the pleiotropic effects of statins, which have proven to be beneficial for the pathophysiology of Chagas cardiomyopathy through their ability to induce the synthesis of endogenous pro-resolutive, anti-inflammatory molecules such as 15-epi-LXA4, which may mediate a decrease in leukocyte adhesion through the inhibition of the NF-κB pathway activation, thus reducing the nuclear migration of p65 [41].

#### 4.3.1. Specialized Pro-Resolving Mediators Mark the Dawn of the Resolution Pharmacology

Initially, during inflammation, prostaglandins E2 and D2 are synthesized from arachidonic acid by the action of cyclooxygenase (COX). When the resolution is activated, the prostaglandins synthesis stops, and a temporal switch leads to the production of the lipoxins (LXs) and their aspirin-triggered analogs (AT-L). LXs were the first discovered SPMs and are known as anti-inflammatory eicosanoids with the ability to retard the infiltration of new neutrophils to inflammation sites, reducing vascular permeability. Then, D-series resolvins (RvD1-6) and protectins (PD and NPD1) are synthesized from docosahexaenoic acid (DHA) by the action of lipoxygenase (LOX) in PMNs and macrophages. The SPM RvD1, Resolvin D2 (RvD2), and Resolvin D5 (RvD5) appear earlier in the resolution phase. RvD2 increases temporally during the transition from initiation to resolution phase with a peak at 6–12 h 27. RvD2 has a greater effect than RvD1 on neutrophils and wound healing [140]. RvD2 is a potent and specific agonist of G protein-coupled receptor 18 (GPR18) in neutrophils, macrophages, and endothelial cells, leading to the activation of the cAMP/PKA/STAT3 signaling pathway and inactivation of the inflammasome, which contributes to a decrease in proinflammatory mediators, PMN adhesion to endothelial cells, and macrophage phagocytosis, with anti-migratory and antiproliferative effects resulting in improved revascularization. Protectins reach their peak at 12 h and have recently been identified as a ligand for G protein-coupled receptor 37 (GPR37) in microglial and T cells, as they regulate the TNFα and INFγ production [141].

E-series resolvins (Resolvin E1 (RvE1), Resolvin E2 (RvE2), and Resolvin E3 (RvE3)) are synthesized from eicosapentaenoic acid (EPA) by the action of 5-lipoxygenase (5-LOX) in PMN and endothelial cells; RvE1 appears at a delayed time point, with peaks between 48 and 72 h [142]. RvE1 is the most effective of the RvE-series in inhibiting neutrophil migration in vitro and promoting wound healing *in vivo* [143] in neutrophils and mucosal epithelial cells. RvE1 binds to Gi/0-coupled Chemerin R23 receptor, leading to the inactivation of cyclic adenosine monophosphate (cAMP), reduction in Ca+2 cytosolic concentration, and inhibition of the translocation of p65-NFκB [144].

Maresins are biosynthesized from DHA via 12-lipoxygenase (12-LOX) by macrophages. The peak of maresins has been observed at 24 h in exudates [145]. It is known that the expression levels of 12-LOX change according to the phenotype of macrophages (M0, M1, M2); thus, the release of maresins polarizes their phenotype from M1 to M2 to exert their biological action on the resolution of inflammation and activation of tissue regeneration [107,146]. Maresin 1 (MaR1) is a selective ligand of leucine-rich repeat-containing G protein-coupled receptor 6 (LGR6) in macrophages, triggering an increase in cAMP, promoting macrophage switch from M1 to M2 and tissue regeneration [147,148].

Resolvins are the most studied SPMs in models of chronic inflammatory diseases, increasing survival in animal models of infection, lung damage, peritonitis, and colitis [149,150,151]. Recently, it was demonstrated that the combined administration of RvE1 and lipoxin A4 resolves the inflammation in a murine model of Alzheimer’s disease, and RvD2 and MaR1 prevent atheroprogression in a murine model, generating a potent anti-inflammatory phenotype and attenuated intima hyperplasia in mice. Interestingly, MaR1 improves efferocytosis in macrophages and has shown a higher potency than RvD1 [152,153,154].

**Figure 3:**
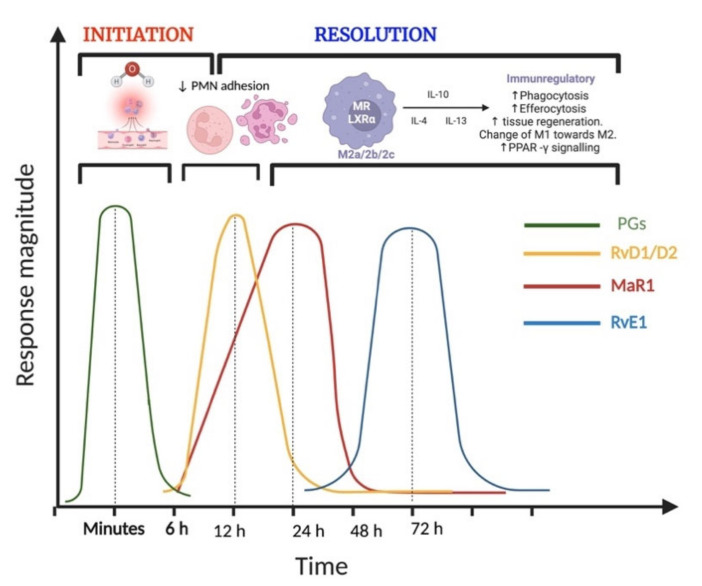
Early and sequential release of specialized pro-resolving mediators in acute inflammation and its resolution. SPMs play an important role in the initiation and resolution of inflammation. Resolvin D2 (RvD2) increases temporally during the transition from initiation to resolution phase with a great effect on neutrophils. Maresin 1 (MaR1) promotes macrophage switch from M1 to M2 after their marked increase. Resolvin E1 (RvE1), the most effective of the RvE-series, inhibits neutrophil migration in vitro and promotes resolution phenotype in macrophages (created with BioRender.com, accessed on 30 July 2022).

SPMs orchestrate the beginning and end of the inflammation resolution (Figure 3) through different biosynthetic routes, target cells, time-dependent release, and biological actions, indicating a constitutive synthesis and differential regulation [154]. Each member of the SPM superfamily is a cardinal point in the resolution of inflammation. Thus, it is possible to find a synergistic action with the combination of SPMs or sequential administration.

#### 4.3.2. Biosynthesis of SPMs

**Figure 4:**
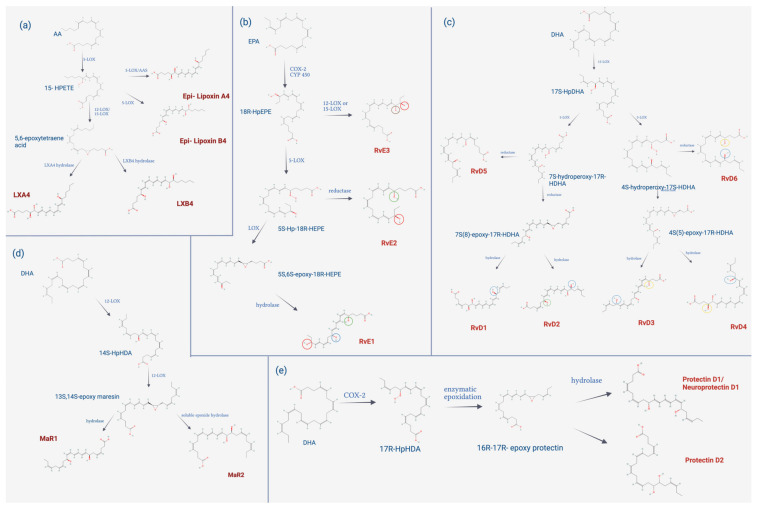
Biosynthesis of SPMs. (**a**) Lipoxin biosynthesis. The precursor of lipoxins is arachidonic acid (AA), which gives rise to lipoxin A4 (LXA4) and lipoxin B4 (LXB4), which are positional isomers, and epi-lipoxin A4 and epi-lipoxin B4, which are more stable than the former. (**b**) Biosynthesis of E-series resolvins (RvE). The precursor of RvE is acid eicosapentaenoic acid (EPA) of 20 carbons. Resolvin E1 (RvE1), resolvin E2 (RvE2), and resolvin E3 (RvE3) are similar in the presence of a hydroxyl group (OH) at carbon 18 (C18) (red circle). RvE1 and RvE2, unlike RvE3, present an OH group at C5 position (green circle). In addition, RvE1 has a third OH group at the C12 position (blue circle). Finally, RvE3 has an OH group in position C17 (brown circle). (**c**) Biosynthesis of D-series resolvins (RvD) RvD originates from the 22-carbon docosahexaenoic acid (DHA). All D-series resolvins have a hydroxyl group (OH) at the C17 position (blue circle). RvD1, RvD2, and RvD5 are generated from the oxygenation of 17S-hydroperoxy-docosahexaenoic acid (17S-HpDHA) in position C7; therefore, these resolvins have in common OH group in the said position (green circle). RvD1 and RvD2, unlike RvD5, are characterized by having a third OH group; RvD1 contains the OH group at the C8 position, while RvD2 has it at the C16 position. On the other hand, RvD3, RvD4, and RvD6 originate from the oxygenation of 17S-HpDHA at the C4 position, therefore these resolvins have a common OH group at that position (yellow circle). RvD3 has a third OH group in the C11 position, while RvD4 has the third OH group in the C5 position. (**d**) Biosynthesis of maresins. The precursor of maresins is DHA originating from two exponents, MaR1 and MaR2. (**e**) Protectin biosynthesis. The precursor of protectins is DHA and originates through enzymatic epoxidation and subsequent hydrolysis of PD1 (created with BioRender.com, accessed on 9 September 2022).

As stated above, SPMs are chemical mediators encompassing different types of molecules including lipoxins, maresins, resolvins, and protectins, which are all biosynthesized from essential fatty acids, which is detailed in Figure 4. Lipoxins are derived from arachidonic acid (AA); their biosynthesis occurs mainly in epithelial cells, vascular endothelium, and leukocytes through the action of lipoxygenases, specifically 15-lipoxygenase (15-LOX).

This enzyme inserts molecular oxygen in the AA at the carbon in position 15, forming 15-hydroperoxy-eicosatetraenoic acid (15-HPETE); this is captured by polymorphonuclear leukocytes and/or monocytes to convert it through 5-LOX into acid 5,6-epoxytetraene, which is then hydrolyzed by the enzyme lipoxin A4 hydrolase or B4 hydrolase, to give rise to lipoxin A4 (LXA4) and lipoxin B4 (LXB4), respectively. However, there is a second biosynthetic pathway, which is dependent on leukotriene A4. This route involves leukocytes and platelets from peripheral blood; the 5-LOX found in leukocytes converts AA into leukotriene A4, and this is taken up by platelets, which, by the action of 12-LOX, gives rise to LXA4 and LXB4 [155,156].

In addition, there is a third biosynthetic pathway that begins with the metabolization of the intermediate 15-HPETE through 5-LOX. From the latter, two lipoxin derivatives arise, 15-epi-LXA4, also called aspirin-activated lipoxin, a drug used as an analgesic, antipyretic, and is currently used for its action of cardiovascular protection and the biosynthesis of 15-epi-lipoxin B4 that is not dependent on aspirin. Additionally, the formation of 15-epi lipoxin has been observed in the presence of statins. Lipoxins, maresins, resolvins, and protectins decrease the activity of soluble epoxide hydrolase, which increases the 14,15-epoxyeicosatrienoic acid that, in turn, influences the conversion to epi lipoxins [157].

Resolvins, on the other hand, are classified into two groups, depending on the type of polyunsaturated fatty acid they come from: the E series, derived from EPA; and the D series, from DHA. E-series resolvins initiate their biosynthesis through the action of acetylated COX-2, which oxygenates EPA, generating 18R-Hydroxyperoxy-eicosapentaenoic acid (18R-HpEPE), then it is reduced to 18R-Hydroxy-eicosapentaenoic acid (18R-HEPE). This intermediate is oxygenated by 5-LOX, forming a hydroperoxide, which can take two routes; if epoxidation occurs followed by enzymatic hydrolysis, it gives rise to RvE1, but if the hydroperoxide is reduced, it gives origin to RvE2. RvE3 is biosynthesized via 12/15-LOX from 18R-HpEPE.

Similarly, the biosynthesis of the D-series resolvins begins through the action of 15-LOX, which oxygenates DHA, giving rise to 17S-Hydroperoxy-docosahexaenoic acid (17S-HpDHA), then the second oxygenation occurs mediated by 5-LOX, originating a hydroperoxide intermediate, which is rapidly reduced to 7S,8S-epoxide, then enzymatic hydrolysis occurs, giving rise to RvD1 and RvD2. 5-LOX catalyzes the oxygenation of 17S-HpDHA at carbon 4, giving rise to RvD3 and resolvin D4 (RvD4). Instead, RvD5 and resolvin D6 (RvD6) are generated by the reduction of 7S- and 4S-hydroxyperoxide, respectively. D-series resolvins and maresins are derived from DHA; their biosynthesis occurs mainly in M2 macrophages and begins through the action of 12-LOX, which inserts molecular oxygen in DHA at carbon position 14, originating 14S-Hydroperoxy-docosahexaenoic acid (14S-HpDHA), which is later transformed into the 13S,14S-epoxide maresin. This intermediate can take different routes; if a hydrolysis reaction of the epoxide occurs, it forms maresin 1.

On the other hand, if the intermediate 13S,14S-epoxide maresin is oxidized to 13R,14S-dihydroxy-docosahexaenoic acid (13R,14S-diHDHA), maresin 2 is formed [158]. There is another route by which maresin 1 is biosynthesized, which occurs through the interaction of platelets and neutrophils, through the action of 12-LOX on platelets, converting DHA into 13S,14S-epoxide maresin, which is subsequently transformed by neutrophils in maresin 1. The protectins, like the previously mentioned SPMs, have DHA as a biosynthetic precursor, which through the action of 15-LOX originates 17S-HpDHA, then through enzymatic epoxidation forms 16S-17S-epoxy-protectin; subsequently, enzymatic hydrolysis occurs, originating protectin D1 (PD1) or neuroprotectin D1 (NPD1), depending on the tissue of biosynthesis [159].

As mentioned above, inflammation is an active, self-limited, and programmed process, which proceeds in highly regulated stages due to the production of endogenous molecules. The initiation is mediated by proinflammatory molecules such as prostaglandins and thromboxane, followed by a switch in the biosynthesis of mediators, allowing for a resolution to take place. Various research models have identified different ways in which these mediators contribute to the resolution of inflammation, recovery of homeostasis, and fostering tissue regeneration.

#### 4.3.3. Biological Functions of SPMs

One of the first mediators to be discovered was lipoxins, which tend to be generated basally in all tissues, through the interaction between platelets, neutrophils, epithelial, and endothelial cells, acting at pico and nanomolar concentrations in tissues, limiting PMN recruitment and activation at the site of inflammation, stimulating efferocytosis as well as monocyte chemotaxis, and promoting the phenotypic shift of macrophages towards M2 and the release of anti-inflammatory cytokines, such as IL-10 and TGF-β [4,159,160].

Two types of lipoxins have been described, LXA4 and LXB4, which are positional isomers that share many biological activities but differ mainly in that LXA4 exerts its action by binding to the G protein-coupled receptor formyl peptide receptor 2 (FPR2/ALX), which is expressed in greater quantities in monocytes, macrophages, epithelial and endothelial cells, while LXB4 has no cognate receptor described.

Resolvins are SPMs that have been extensively investigated. Regarding the E series, it has been demonstrated that RvE1 inhibits the release of interleukin-23 (IL-23) and decreases the production of interleukin-12 (IL-12) in DC; it also increases the expression of the receptor 5 chemokine cysteine–cysteine cysteine (CCR5) that contributes to the mobility of leukocytes including T lymphocytes [160]. RvE2 has been shown to decrease neutrophil chemotaxis and stimulate efferocytosis and phagocytosis of apoptotic neutrophils and bacteria. Both RvE1 and RvE2 have been described to activate chemerin receptor 23 (ChemR23), which is expressed in monocytes, macrophages, and dendritic and myeloid cells; it also interacts as a partial agonist with leukotriene B4 receptor 1 (BLT1). With respect to RvE3, it showed a greater decrease in IL-23 release compared to the use of RvE1 and RvE2.

Regarding D-series resolvins, it has been shown that both RvD1 and RvD2 stimulate phagocytosis of apoptotic neutrophils, RvD3, in addition to decreasing neutrophil migration, decreasing the release of proinflammatory cytokines such as IL-6 and IL-23 and promotes macrophage efferocytosis [161]. RvD1, as well as RvD3, activate the FPR2/ALX receptor [162]. In contrast, RvD2 interacts with the GPR18, which is abundantly expressed on monocytes, macrophages, and neutrophils. This interaction has been shown to have a protective role against sepsis, as it favors bacterial phagocytosis [163]. RvD1, RvD3, and RvD5 also activate G protein-coupled receptor 32 (GPR32).

In recent years, through various investigations, the actions of maresin in the resolution of inflammation have been discovered through the binding and activation of LGR6, which is highly expressed in phagocytes, where it has been shown to stimulate phagocytosis and spherocytosis, decrease PMN infiltration, and increase tissue regeneration, and one of the actions that has been most emphasized for its possible therapeutic role is its ability to reduce neuropathic pain [164]. Two exponents have been described, MaR1 and maresine 2 (MaR2), which act at peak and nanomolar levels; both have similar biological actions, but it has been shown that MaR2 is less potent in capturing apoptotic PMN compared to MaR1 [163].

Finally, there is the action of the mediator PD1, which has been shown to inhibit PMN leukocyte infiltration and inhibit macrophage efferocytosis, decrease TNF-α and IL-6 expression, and increase IL-10 release, but one of the roles that has been most investigated is its neuroprotective role, associated more with NPD1, which differs from PD1 by the origin of its biosynthesis. Table 2 summarizes the evidence found on the cell targets, receptors, and biological effects of each of the SPMs. It is noteworthy that RvE1, LXA4, RvD2, and MaR1 are the most studied, and therefore, the mechanism and biological effect are better known.

#### 4.3.4. Limitations and Future Directions in Resolution Pharmacology

Undoubtedly, research has yielded data that demonstrate the fundamental role of SPMs in the resolution of inflammation, generating great hope for their therapeutic applicability in different pathologies, but for this, it is essential to know the pharmacokinetic properties of these molecules. For example, lipoxins are produced endogenously in most tissues but are subject to rapid catalysis mediated by the enzyme prostaglandin dehydrogenase (PGDH) and subsequent inactivation by an ω-oxidoreductase, which has greatly limited their direct therapeutic applicability. However, it has been found that epi-LXA4 and epi-LXB4 analogs are more resistant to enzymatic transformation, so adding functional groups to the structure can improve the stability, thus preventing PGDH action. In this way, synthetic analogs were developed which have shown similar actions and higher oral bioavailability, but they are still susceptible to beta-oxidation, decreasing the time of their availability in plasma [167].

The first generations of synthetic analogs protected the hydroxyl groups with acetate groups to delay their metabolic inactivation. Currently, the fourth generation of analogs is being developed, the most promising being the benzo-lipoxin A4 (benzo-LXA4) derivatives, wherein the tetraene of LXA4 is replaced by a ring fused by a benzene, providing greater thermal stability to the molecule and better resistance to enzymatic inactivation, in an easy-to-synthesize manner [157]. In this way, the main problems were solved and even with an improved potency, which allows using much lower concentrations to generate the same actions.

Regarding the resolvins, something similar occurs: both the E and D series are susceptible to eicosanoid oxidoreductase (EOR) enzymes that rapidly inactivate them; in fact, the enzyme 15-prostaglandin dehydrogenase (15-PGDH) in the presence of the cofactor NADH rapidly metabolizes RvD1, approximately in 25 min, generating two metabolites, 8-oxoresolvin D1 (8-oxo-RvD1) and 17-oxoresolvin D1 (17-oxo-RvD1), the former having a similar activity in reducing neutrophil infiltration, while the latter is considered inactive since its biological activity is drastically reduced [165].

The position of the substituents also affects the stability; one study found that the R epimer is less susceptible to metabolization than the S epimer, which results in a more prolonged action [164]. In addition, Rvs receptors have been shown to be stereoselective, so it is also important to know the impact of the enantiomer geometry and stereochemistry on their activity.

Regarding the kinetics of resolvin release, an in vivo investigation of a *Staphylococcus aureus* infection has shown that RvD1 and RvD2 are released in early stages, with a peak of maximum concentration 12 h after the onset of infection. On the other hand, RvD3 and RvD4 are released late; RvD3 presents low levels between 0 and 48 h, but begins to increase after 48 h, having a peak at 72 h; and RvD4 levels are maintained until 72 h after the beginning of the infection [159,168]. In addition to the metabolic instability of these mediators, which makes them have a short half-life, they show low solubility in water, and they are chemically unstable since they are labile to light and temperature. All these characteristics are limitations that have added to their possible exogenous administration since the bioavailability of native resolvins would be very low. For this reason, in recent years, synthetic analogs have been developed to overcome the main limitations.

The 19-(9-fluorophenoxy)-resolvin E1 analog was developed, which resists metabolic inactivation by PGDH and retains biological activity, such as reducing PMN infiltration and decreasing the synthesis of proinflammatory cytokines [169]. Another example of this is the methyl ester analog of benzo-diacetylene-17R-RvD1 (BDA-RvD1) which is much more resistant to the action of 15-PGDH, as it is not converted to the inactive metabolite, 17-ox-RvD1 [170]. Another relevant discovery is that aspirin-activated mediators are metabolized much more slowly, indicating the stereoselectivity of the 15-PGDH enzyme.

In addition to synthetic analogs, other strategies have been developed to solve these problems, through different dosage forms, such as the use of liposomes and nanoparticles. A recent study encapsulated an analog of RvD1 that allowed increasing tissue selectivity and circulation times using targeting ligands and polyethylene glycol. These products have been shown to decrease the levels of proinflammatory cytokines and the infiltration of neutrophils in the lungs [171]. The information about the pharmacokinetics of maresins and protectins has not yet been investigated. It is only known that they tend to be enzymatically unstable [165]. However, despite these limitations, they continue to demonstrate pro-resolution efficacy without the associated toxicity. Indeed, SPMs have shown a role in wound healing. Thus, RvE1 has been shown to promote the migration of keratinocytes and reduce inflammation, and RvD2 has also been shown to promote keratinocytes migration but reduces fibroblast proliferation and migration. For LXA4, the capacity to induce fibroblasts migration and proliferation has been reported. Finally, for maresin, a role in macrophage polarization and inflammation reduction has been indicated [172]. Therefore, these molecules play a role not deeply examined yet, in the resolution of inflammation needed in the wound healing process.

## 5. Concluding Remarks

Inflammation is a double-edged sword. On the one hand, it is necessary to destroy pathogens and to control sterile injuries; on the other hand, when the process is not resolved, it affects homeostasis and tissue regeneration. The latter is important as unresolved inflammation may lead to several important chronic diseases that place a burden on modern public health. Specialized pro-resolving mediators, including lipoxins, maresins, resolvins, and protectins, play many important roles in the restoration of the balance in several physiopathological conditions associated with unlimited inflammation. In this review, we have described the role of inflammation in the acute and chronic response to pathogen infection and the inflammatory reaction to adjuvants in vaccine formulations, and we have established that unresolved inflammation is involved in the pathophysiology of chronic diseases. We reviewed the synthesis and function of the most prominent SPMs, and we introduced resolution pharmacology. Finally, the role of advanced therapies for chronic wound resolution includes tissue engineering approaches that investigate scaffolds, which mimic the extracellular matrix, promoting wound healing through the secretion of trophic and anti-inflammatory mediators by the attached cells. In the future, early detection of clinical biomarkers for chronic inflammation and the detection of low levels of SPMs will aid in avoiding progression to chronic diseases and inflammatory damage. Pharmaceutical development of SPMs into stable dosage forms will open up exciting alternatives for human therapeutics.

## Figures and Tables

**Table 1:** Representative clinical trials in course for cell therapy and tissue engineering for chronic wounds *.

Type of Cells	Phase	Sponsor	Description
Stromal fraction	I	Antria	Stromal Vascular Fraction from Lipoaspirate in the Treatment of Chronic Non-healing Wounds
SkinTE: keratinocytes, dermal fibroblasts, dermal endothelial cells, and follicular cells, as well as extracellular matrix.	III	PolarityTE	Multi-Center, Prospective, Randomized Controlled Trial Evaluating SkinTE® in the Treatment of Wagner 2 Diabetic Food Ulcer
ABCB5-positive dermal mesenchymal stromal cells	IIB	RHEACELL GmbH & Co. KG	Allogeneic ABCB5-positive Dermal Mesenchymal Stromal Cells for Treatment of Chronic Venous Ulcers
Mesenchymal stem cells	I	University of Colorado, Denver	Open-Label Safety Study of Umbilical Cord Lining Mesenchymal Stem Cells (Corlicyte®) To Heal Chronic Diabetic Foot Ulcers
Mesenchymal stem cells	II	Instituto para el Desarrollo Biotecnológico y la Innovación	Cell Therapy for Diabetic Foot Ulcer
Umbilical cord blood mononuclear cells	III	Peking University Third Hospital	Efficacy and Safety of Umbilical Cord Blood Mononuclear Cell Gel in the Treatment of Refractory Diabetic Foot Ulcer.
Mesenchymal stem cells	I	Beijing Tongren Hospital	Human Placental Mesenchymal Stem Cells Treatment on Diabetic Foot Ulcer
Mesenchymal stem cells	II	Anterogen Co., Ltd.	Efficacy and Safety of ALLO-ASC-SHEET in Subjects with Diabetic Foot Ulcers. It is a hydrogel with allogenic adipocytes-derived mesenchymal cells

* built with data retrieved from clinicaltrials.gov using stem cells and chronic wounds as search terms.

**Table 2:** Receptors, target cells, and biological function of SPMs.

Ligands	Receptors	Target cells	Function	References
RvE1	ChemR23 BLT1	Monocytes, macrophages, dendritic cells and myeloid cells.	↑ phagocytosis by macrophages. Limits neutrophil signals. ↑ PMN apoptosis. ↓ IL-12 production. It inhibits the release of IL-23. ↑ expression of CCR5 that contributes to the mobility of leukocytes including T cells.	[165]
RvE2	ChemR23 BLT1	Monocytes, macrophages, dendritic cells and myeloid cells.	↓ neutrophil chemotaxis. ↑ phagocytosis of apoptotic neutrophils. ↑ phagocytosis of bacteria. ↑ efferocytosis.	[146]
RvE3	---	Neutrophils	Stops neutrophil chemotaxis. ↓ IL-4, IL-5, IL-13, IL-17, and IL-23 mRNA levels in lung cell culture.	[146]
RvD1	GPR32 (↑ affinity) ALX/FPR2	Leukocyte and macrophages.	↑ phagocytosis of macrophages and apoptotic leukocytes. ↑ efferocytosis. ↑ polarization of M2 macrophages.	[157]
RvD2	GRP18	Monocytes, macrophages and neutrophils.	↑ phagocytosis of apoptotic neutrophils. ↓ movement of neutrophils ↑ efferocytosis. Protective role against sepsis, since it favors phagocytosis of bacteria.	[166]
RvD3	ALX/FPR2 GPR32	Monocytes, macrophages and neutrophils.	↑ phagocytosis of apoptotic cells ↓ migration of neutrophils. ↑ efferocytosis. ↓ release of IL-6 and IL-23. ↑ IL-10 levels.	[164]
RvD4	---	Neutrophils.	↓ neutrophil infiltration. ↑ efferocytosis. It inhibits the production of cytokines. Regulates diapedesis of leukocytes.	[159]
RvD5	GPR32	Macrophages.	↑ phagocytosis by macrophages.	[166]
LXA4	ALX/FPR2 GPR32	Monocytes, macrophages, epithelia, endothelia, and fibroblasts.	They limit neutrophil recruitment and PMN activation. They promote the chemotaxis of monocytes and their infiltration in infectious foci. ↑ efferocytosis. ↑ phenotypic shift of macrophages towards M2. ↑ release of IL-10 and TGF-β.	[167]
LXB4	---	---	Biological activity very similar to that of LXA4.	[167]
MaR1	LGR6 BLT1 (↓ affinity)	Macrophages, monocytes, and PMNs.	↑ phagocytosis ↑ efferocytosis Regulates PMN chemotaxis. ↓ neuropathic pain. ↑ tissue regeneration. ↓ expression of IL-5 and IL-13. ↓ proliferation, migration and differentiation of fibroblasts.It stimulates phenotypic change of macrophages towards M2.	[147,168]
MaR2	---	Macrophages, monocytes, and PMNs.	↑ phagocytosis. ↓ PMN infiltration.	[162,166]
PD1	---	Leukocytes, macrophages and neutrophils.	Inhibits infiltration of polymorphonuclear leukocytes. ↓ expression of TNF-α and IL-6. ↑IL-10 release.	[169]

---: Indicates that no information has been described in the literature.

## Data Availability

Not applicable.

## Abbreviations

12-LOX	12-Lipoxygenase
13R,14S-diHDHA	13R,14S-dihydroxy-docosahexaenoic acid
14S–HpDHA	14S-hydroperoxy-docosahexaenoic acid
15-epi-LXA4	15-epi-lipoxin A4
15-HPETE	15-hydroperoxy-eicosatetraenoic acid
15-LOX	15-lipoxygenase
15-PGDH	15-prostaglandin dehydrogenase
17-oxo-RvD1	17-oxoresolvin D1
17S-HpDHA	17S-hydroperoxy-docosahexaenoic acid
18R-HEPE	18R-hydroxy-eicosapentaenoic acid
18R-HpEPE	18R-hydroxyperoxy-eicosapentaenoic acid
5-LOX	5-lipoxygenase
8-oxo-RvD1	8-oxoresolvin D1
AA	arachidonic acid
APC	antigen-presenting cell
ARG	arginase
AT-L	aspirin-triggered analogs
BDA-RvD1	benzo-diacetylene-17R-RvD1
benzo-LXA4	benzo-lipoxin A4
bFGF	basic fibroblast growth factor
BLT1	leukotriene B4 receptor 1
CAD	coronary artery disease
cAMP	cyclic adenosine monophosphate
CAMs	cell adhesion molecules
CANTOS	canakinumab anti-inflammatory thrombosis outcomes study
CCL2	chemokine C-C motif ligand 2
CCL20	chemokine C-C motif ligand 20
CCL3	chemokine C-C motif ligand 3
CCL4	chemokine C-C motif ligand 4
CCL8	chemokine C-C motif ligand 8
CCR5	receptor 5 chemokine cysteine-cysteine cysteine
CD105	cluster of differentiation 105
CD11b	cluster of differentiation 11b
CD14	cluster of differentiation 14
CD19	cluster of differentiation 19
CD34	cluster of differentiation 34
CD73	cluster of differentiation 73
CD79α	cluster of differentiation 79 α
CD80	cluster of differentiation 80
CD83	cluster of differentiation 83
CD86	cluster of differentiation 86
CD90	cluster of differentiation 90
ChemR23	chemerin receptor 23
CIRT	cardiovascular Inflammation Reduction Trial
COX	cyclooxygenase
COX-2	cyclooxygenase-2
CRP	C-reactive protein
CRS	Cytokine Release Syndrome
CVDs	cardiovascular diseases
CXCL1	chemokine C-X-C motif ligand-1
CXCL2	chemokine C-X-C motif ligand-2
CXCL5	chemokine C-X-C motif ligand-5
CYP	cytochrome
CYP1A	cytochrome P450 1A
CYP2B1/2	cytochrome P450 2B1/2
CYP3A4	cytochrome P450 3A4
DAMPs	damage associated molecular patterns
DCs	dendritic cells
DHA	docosahexaenoic acid
E-CAMs	extracellular matrix adhesion molecules
ECM	extracellular matrix
EOR	eicosanoid oxidoreductase
EPA	eicosapentaenoic acid
ET-1	endothelin-1
FDA	food and drug administration
FGF	fibroblast growth factor
FPR2/ALX	formyl peptide receptor 2
FPR2/ALX	formyl peptide receptor 2
GPR18	G protein-coupled receptor 18
GPR32	G protein-couple receptor 32
GPR37	G protein-coupled receptor 37
HLA DR	human leukocyte antigen—DR isotype
IBS	innate immune system
ICAM-1	intercellular adhesion molecule 1
IFN	interferon
IFN-α/β	interferons alpha/beta
IFN-β	interferon β
IFN- γ	interferon γ
IL-1	interleukin-1
IL-1β	interleukin-1 β
IL-10	interleukin-10
IL-12	interleukin-12
IL-13	Interleukin-13
IL-17β	interleukin- 17 β
IL-2	interleukin-2
IL-23	interleukin-23
IL-6	interleukin-6
IRF7	interferon regulatory factor 7
LDL	low density lipoprotein
LGR6	G protein-coupled receptor 6
LOX	lipoxygenase
LPS	lipopolysaccharide
LXA4	lipoxin A4
LXB4	lipoxin B4
LXs	lipoxins
MAIT	mucosal Associated Innate T cells
MaR1	maresin 1
MaR2	maresine 2
MCP-1	monocyte chemoattractant protein-1
MHC	major histocompatibility molecules
miRNA	microRNA
MPL	monophosphoryl lipid A
MSCs	mesenchymal stem cells
MyD88/RAK/TRAF6	myeloid differentiation factor 88/IL-1 receptor-associated kinase 1/tumor necrosis factor receptor-associated factor 6
MyD88/TIRAP	myeloid differentiation factor 88/domain-containing adaptor protein
NF-κB	nuclear factor kappa B
NLRs	nod-like receptors
NO	nitric oxide
NOTCH-1	neurogenic locus notch homolog protein 1
NPD1	neuroprotectin D1
PAMPs	pathogen associated molecular patterns
PD1	protectin D1
PDGF	platelet-derived growth factor
PECAM-1	platelet endothelial cell adhesion molecule-1
PGDH	prostaglandin dehydrogenase
PGI2	prostacyclin
PMN	polymorphonuclear neutrophils
PRRs	pattern recognition receptors
ROS	reactive oxygen species
RvD1	resolvin D1
RvD1-6	D-series resolvins
RvD2	resolvin D2
RvD4	resolvin D4
RvD5	resolvin D5
RvE1	resolvin E1
RvE2	resolvin E2
RvE3	resolvin E3
SIE	specific immune system
SPM	specialized pro-resolving
*T cruzi*	*Trypanosoma cruzi*
TGF-β	transforming growth factor β
TGF-β3	transforming growth factor β3
TLR2	toll-like receptor 2
TLR4	toll-like receptor 4
TLRs	toll-like receptors
TNFα	tumor necrosis factor alpha
TRIF/TRAM	TIR-domain-containing adapter-inducing interferon-β/TRIF-related adaptor molecule
TSG-6	tumor necrosis factor alfa stimulated gene 6
TXA2	thromboxane A2
VCAM-1	vascular cell adhesion molecule-1
VEGF	vascular endothelial growth factor
VSMCs	vascular smooth muscle cells
